# Efficient Photocatalytic Removal of Aqueous Ammonia Nitrogen by g-C_3_N_4_/CoP Heterojunctions Under Visible Light Illumination

**DOI:** 10.3390/nano14241996

**Published:** 2024-12-13

**Authors:** Dongxu Wang, Wanfeng Mao, Lihong Zhao, Duo Meng, Jiaqi Tang, Tengfei Wu

**Affiliations:** 1 Department of Civil and Architecture Engineering, Liaoning University of Technology, Jinzhou 121000, China; 2 Jinzhou Water Affairs (Group) Co., Ltd., Jinzhou 121000, China; 3 Institute of Agricultural Resources and Environment, Guangdong Academy of Agricultural Sciences, Guangzhou 510640, China

**Keywords:** g-C_3_N_4_/CoP heterojunction, ammonia nitrogen removal, photocatalytic technology, nitrogen selectivity

## Abstract

With the development of industry, agriculture, and aquaculture, excessive ammonia nitrogen mainly involving ionic ammonia (NH_4_^+^) and molecular ammonia (NH_3_) has inevitable access to the aquatic environment, posing a severe threat to water safety. Photocatalytic technology shows great advantages for ammonia nitrogen removal, such as its efficiency, reusability, low cost, and environmental friendliness. In this study, CP (g-C_3_N_4_/CoP) composite materials, which exhibited high-efficiency ammonia nitrogen removal, were synthesized through a simple self-assembly method. For the optimal CP-10 (10% CoP) samples, the removal rate of ammonia nitrogen reached up to 94.8% within 80 min under visible light illumination. In addition, the nitrogen selectivity $S\left(N_{2}\right)$ is about 60% for all oxidative products. The high performance of the CP-10 photocatalysts can be ascribed to the effective separation and transmission of electron–hole pairs caused by their heterogeneous structure. This research has significance for the application of photocatalysis for the remediation of ammonia nitrogen wastewater.

## 1. Introduction

Ammonia nitrogen as the final product of protein catabolism and metabolism is composed of ionic ammonia (NH_4_^+^) and molecular ammonia (NH_3_), and its concentration is usually lower than 0.2 mg/L in naturally occurring water [1,2]. However, with the rapid development of agriculture, industry, and aquaculture, the excessive concentrations of ammonia nitrogen (>500 mg/L) have regularly been found in aquatic environments, contributing to water eutrophication and threatening the safety of aquatic ecosystems [3,4]. Traditional methods, including physical–chemical and biological methods, such as stripping, adsorption, ion exchange, reverse osmosis, and biological denitrification, have been utilized to eliminate ammonia nitrogen in aquatic environments [2,5]. It is noteworthy that some deficiencies of traditional methods have also been found during the process of operation. For example, the effect of biological treatment is closely related to the pollutant concentration, which is usually used to treat low-concentration ammonia nitrogen [2]. Furthermore, traditional methods also present issues related to secondary pollution, low removal efficiency, and high cost [6]. Therefore, it is necessary to seek an appropriate ammonia nitrogen degradation technology to overcome the defects of traditional methods.

In recent decades, photocatalysis as a specific chemical method has been widely studied in the fields of water splitting, carbon dioxide reduction, disinfection, and the degradation of various water pollutants due to its great advantages in terms of efficiency, reusability, cost, and environment friendliness [7,8]. In the photocatalytic process, semiconductors transfer light energy to chemical energy, resulting in a large number of holes and reactive oxygen species with high oxidation capacity, which can achieve the purpose of pollutant treatment. As for ammonia nitrogen pollution, ammonia can be converted to nonpolluting nitrogen through photocatalytic technology [9]. To date, an abundance of semiconductors, such as TiO_2_, ZnO, Fe_3_O_4_, ZnFe_2_O_4_, ZnSe/BiVO_4_, RuO_2_/TiO_2_, and Cu/ZnO/rGO semiconductors, have been explored as photocatalysts for ammonia nitrogen removal [3,5,10]. These nanoparticles have the advantages of non-toxicity, wide distribution, easy synthesis, and low cost, but the potential release of metal ions from metal-containing semiconductors has raised concerns about secondary pollution [11,12]. Thus, it is necessary to develop efficient metal-free photocatalysts for the degradation of ammonia nitrogen.

Graphitic carbon nitride (g-C_3_N_4_), as a reliable polymeric photocatalyst, was first used for photocatalytic hydrogen production by Wang et al. in 2009 [13]. Subsequently, g-C_3_N_4_ has been widely investigated in the fields of pollutant degradation, water disinfection, carbon dioxide reduction, and electrode construction due to its abundant precursors, simple synthesis method, suitable band gap, and chemical stability [14]. However, the catalytic activity of g-C_3_N_4_ for the degradation of ammonia nitrogen is restricted by the separation efficiency of photo-generated electron–hole pairs and the property of light response [15]. To date, several strategies have been applied to enhance the photocatalytic performance of g-C_3_N_4_ for the degradation of ammonia nitrogen, including structural modification, doping, heterojunction construction, and the addition of co-catalysts [14]. Wang et al. prepared graphene quantum dot modified g-C_3_N_4_ photocatalysts to treat ammonia nitrogen, and the results showed that the removal rate of total ammonia nitrogen reached 90% in 7 h under visible light illumination [16]. As mentioned above, efforts have been made to modify g-C_3_N_4_ photocatalysts in order to promote the elimination of ammonia nitrogen. However, there is still room to develop the photocatalytic efficiency of g-C_3_N_4_ photocatalysts for ammonia nitrogen removal. Cobalt phosphide (CoP), with high stability, low overpotentials, and environmental friendliness, has been reported as an efficient co-catalyst [17]. In previous studies, CoP showed excellent electron capture capability, which was applied to hydrogen evolution [18]. It is noteworthy that the CoP/g-C_3_N_4_ heterojunction with outstanding photoelectrochemical properties garnered great attention due to its potential in the fields of pollutant removal, CO_2_ reduction, hydrogen peroxide production, and water splitting. For example, Zhang et al. prepared CoP/g-C_3_N_4_ photocatalysts, which exhibited excellent photocatalytic activity in water hydrogen evolution [19]. Kaito et al. synthesized a co-doped CoP/g-C_3_N_4_ composite, and the H_2_O_2_ production rate reached up 293% [20]. Zhang et al. successfully obtained CoP/Fe_2_O_3_/g-C_3_N_4_ samples, which not only enhanced hydrogen evolution but also increased CO_2_ conversion [21]. Nonetheless, there are few reports on CoP/g-C_3_N_4_ photocatalysts for the degradation of ammonia nitrogen.

Herein, the strategy of noble-metal-free CoP modified g-C_3_N_4_ was employed to prepare CoP/g-C_3_N_4_ composites, and their photocatalytic performance in the degradation of ammonia nitrogen was further evaluated. Compared with pure g-C_3_N_4_, the CoP/g-C_3_N_4_ photocatalysts obtained in this study presented greater ammonia nitrogen elimination activity. This research is regarded to be promising for promoting the application of photocatalysis in the field of ammonia nitrogen elimination.

## 2. Experimental Section

### 2.1. Materials and Reagents

Dicyandiamide, sodium citrate, and sodium hypophosphite hydrate were purchased from Aladdin. Cobalt nitrate hexahydrate (Co(NO_3_)_3_·6H_2_O, AR), sodium hydroxide (NaOH, AR), and ammonium chloride (NH_4_Cl) were obtained from Sinopharm Chemical Reagent Co., Ltd. (Shanghai, China). Nessler’s reagent was purchased from Macklin.

### 2.2. Preparation of g-C_3_N_4_ and CoP Composites

g-C_3_N_4_ was synthesized through the typical thermal method. Briefly, 3 g of dicyandiamide was added into a crucible and then heated in a muffle furnace at 550 °C for 3 h with a heating rate of 5 °C/min. The resultant product was ground into powder to obtain the g-C_3_N_4_ photocatalysts. CoP was obtained through the method reported in our previous work [22].

### 2.3. Preparation of CoP/g-C_3_N_4_ Photocatalysts

CoP/g-C_3_N_4_ photocatalysts were fabricated using the self-assembly method. First, a certain amount of CoP samples was added into 30 mL of anhydrous ethanol, and the mixture was stirred for 5 min. Then, 0.1 g of the prepared g-C_3_N_4_ was dispersed into the above mixtures and persistently stirred for 30 min to obtain a mixture solution. Finally, the mixture solution was treated with an oil bath at 80 °C to evaporate nearly all anhydrous ethanol, and the obtained composites were dried at 60 °C. The prepared CoP/g-C_3_N_4_ photocatalyst was named CP-x for short, where x (x = 1, 2, 3, 10, or 20) represents the various weight percentages of CoP.

### 2.4. Characterization

The X-ray diffraction (XRD) patterns of prepared photocatalysts were obtained using a Rigaku diffractometer. The morphology and the microstructure were analyzed through transmission electron microscopy (TEM, Talos F200X, MA, USA). X-ray photoelectron spectroscopy (XPS) analysis was obtained using the X-ray photoelectron spectrometer (Thermo EscaLab 250Xi, MA, USA). The UV-vis spectra were collected using a UV-vis spectrophotometer (Shimadzu, UV3600i Plus, Kyoto, Japan). The photoelectric current, electrochemical impedance spectroscopy (EIS), and Mott–Schottky analysis were carried out using a CS310H electrochemical station with a normal three-electrode system (CorrTest Instruments, Hubei, China). The active species of the prepared photocatalysts was measured through electron spin resonance (ESR, EMX Pius, Fellanden, CH).

### 2.5. Photocatalytic Experiment

The photocatalytic degradation of ammonia nitrogen was carried out in a self-established photocatalytic reactor. The reactor mainly consists of a 300 W xenon lamp with a UV filter (Zhongjiaojinyuan, CEL-HXUV300, λ > 400 nm), a magnetic stirrer, and a circulatory cooling system. The photocatalytic ammonia nitrogen removal process is described as follows. Firstly, ammonia nitrogen was obtained using ammonium chloride, and the concentration was 30 mg/L. The pH of ammonia nitrogen was 12, which was adjusted using sodium hydroxide. During the experiment process, 25 mg of photocatalysts was mixed with 50 mL of ammonia nitrogen solution, and the mixture solution was stirred in the dark for 30 min to achieve adsorption–desorption balance. Afterwards, a photocatalytic process was performed through the photocatalytic reactor, and the reaction time was 80 min. Then, 2 mL of the mixture solution was collected and filtered using a 0.22 μm membrane. Then, the concentration of ammonia nitrogen was measured using a spectrophotometer.

In this research, the radical trapping experiments were used to identify the contribution of ROS for ammonia nitrogen removal. The trapping agents were added in the dark reaction, and the other operation is consistent with the degradation of ammonia nitrogen. In addition, h^+^, ·O^2−^, and ·OH were quenched through Ethylenediaminetetraacetic acid disodium salt (EDTA-2Na), L-ascorbic acid (LA), and isopropanol (IPA), respectively.

### 2.6. Detection of Ammonia Nitrogen and Total Nitrogen

Nessler’s reagent spectrophotometry and alkaline potassium persulfate digestion ultraviolet spectrophotometry were used to investigate the concentration of ammonia nitrogen and total nitrogen, respectively [5,23]. The removal rate of ammonia nitrogen and nitrogen selectivity $S\left(N_{2}\right)$ can be obtained using the following Equation:

(1) $$\mathrm{Removal}\;({\mathrm{NH}}_{4}^{+}/{\mathrm{NH}}_{3})=\frac{C_{t}\left({\mathrm{NH}}_{4}^{+}/{\mathrm{NH}}_{3}\right)}{C_{0}\left({\mathrm{NH}}_{4}^{+}/{\mathrm{NH}}_{3}\right)}\times 100\%$$

(2) $$S\left(N_{2}\right)=\frac{C_{0}\left({\mathrm{NH}}_{4}^{+}/{\mathrm{NH}}_{3}\right){-\;C}_{t}\left(\mathrm{TN}\right)}{C_{0}\left({\mathrm{NH}}_{4}^{+}/{\mathrm{NH}}_{3}\right){-\;C}_{t}\left({\mathrm{NH}}_{4}^{+}/{\mathrm{NH}}_{3}\right)}\times 100\%$$

where $C_{0}\left({\mathrm{NH}}_{4}^{+}/{\mathrm{NH}}_{3}\right)$ is the concentration of ammonia nitrogen at time 0 and $C_{t}\left({\mathrm{NH}}_{4}^{+}/{\mathrm{NH}}_{3}\right){\;\mathrm{and}\;C}_{t}\left(\mathrm{TN}\right)$ are the concentration of ammonia nitrogen and inorganic total nitrogen at the time t, respectively.

## 3. Results and Discussion

### 3.1. Characterization of Photocatalysts

**Figure 1:**
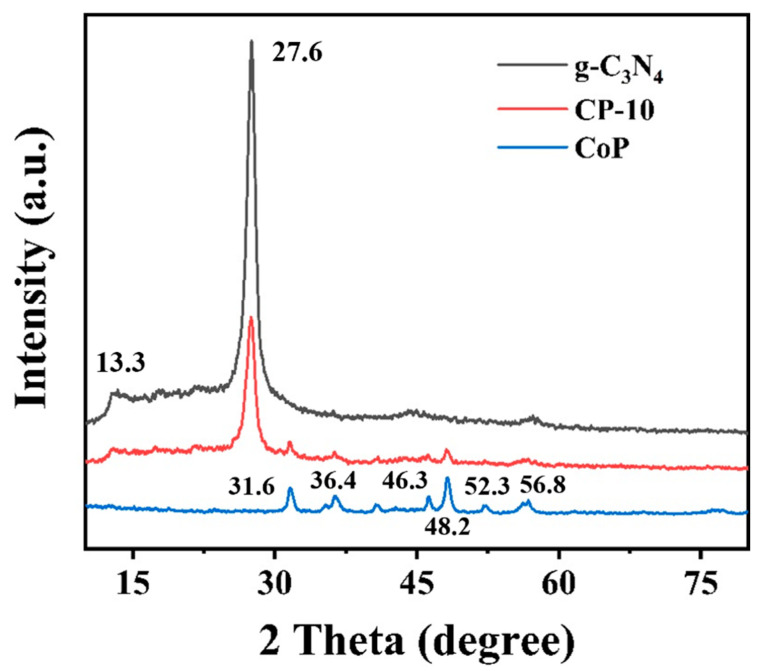
XRD patterns of synthesized photocatalysts.

XRD patterns were used to analyze the crystalline structures of the prepared photocatalysts. As shown in Figure 1, pure g-C_3_N_4_ photocatalysts exhibited obvious diffraction peaks at 13.3° (100) and 27.6° (002), which can be ascribed to in-plane structural packing and interlayer stacking of aromatic systems [24]. The obvious characteristic peaks of pure CoP can be observed at 31.6° (011), 36.4° (111), 46.3° (112), 48.2° (211), 52.3° (103), and 56.8° (301), which are in good accordance with the JCPDS NO. 29-0497 [17]. For CoP/g-C_3_N_4_ photocatalysts, all characteristic peaks were consistent with those of the pure g-C_3_N_4_ and CoP samples, and no impurity peak was detected. The results indicated that the composite photocatalysts were successfully synthesized.

**Figure 2:**
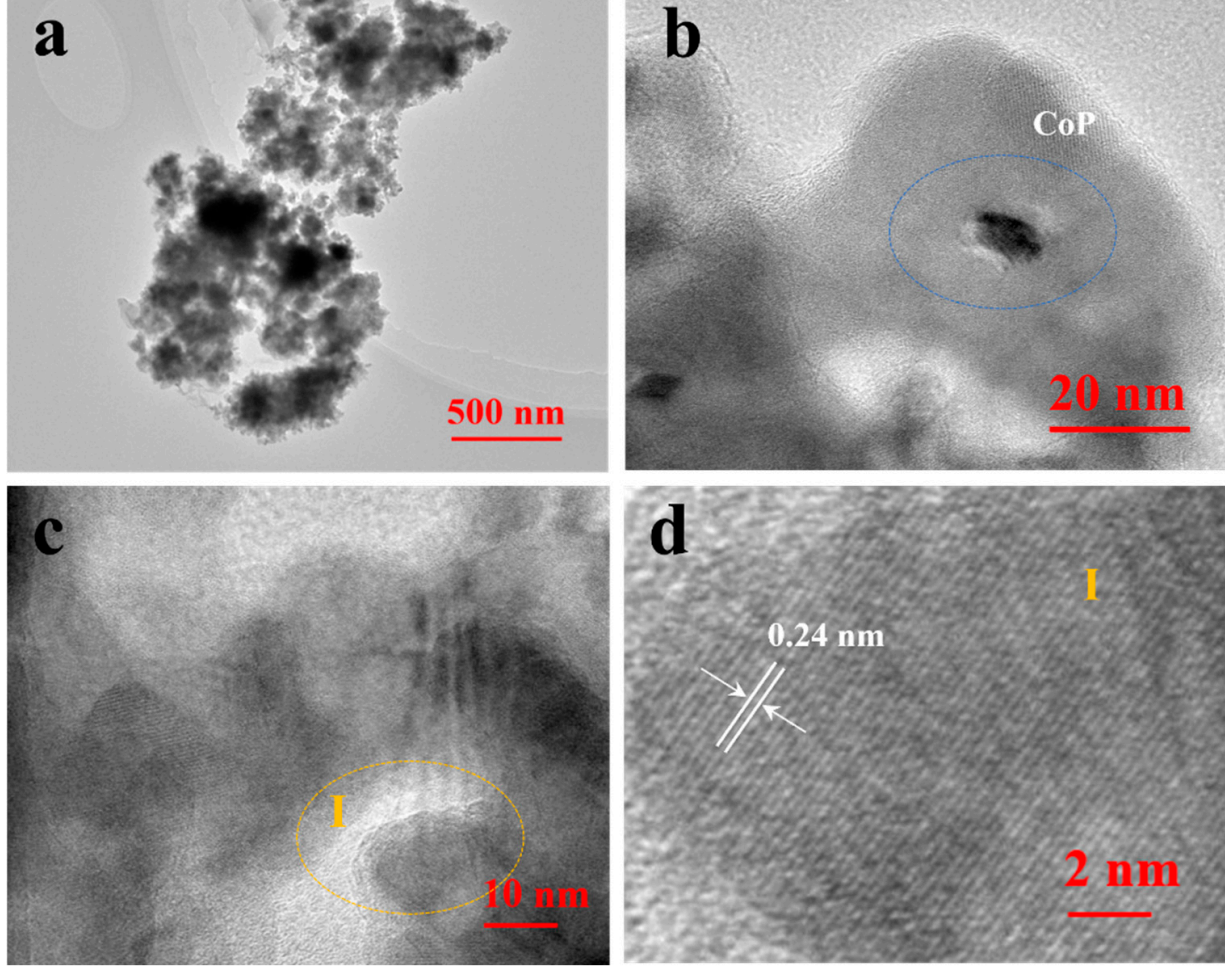
(**a**) TEM image; (**b**–**d**) HRTEM images of CoP/g-C_3_N_4_.

The TEM and HRTEM were used to observe the morphologies of the prepared CoP/g-C_3_N_4_. As shown in Figure 2a, the samples exhibited a bulk structure with a stacked layer, which could be attributed to the skeleton structure of g-C_3_N_4_. It was observed that CoP was bound onto the surface of the g-C_3_N_4_ (Figure 2b). Additionally, HRTEM (Figure 2c,d) indicated the clear lattice fringes of CoP, where the lattice spacing was 0.24, corresponding to the (102) plane of the CoP [25].

**Figure 3:**
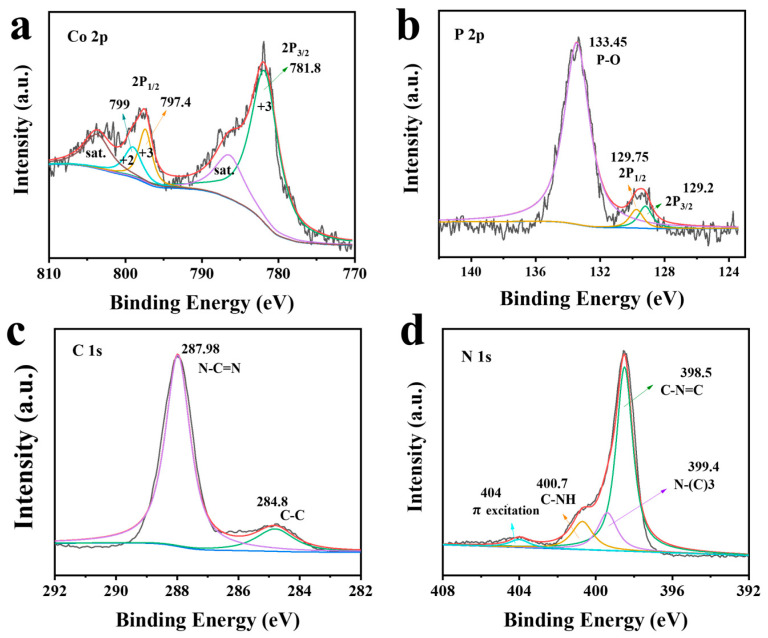
(**a**) Co 2p, (**b**) P 2p, (**c**) C 1s, and (**d**) N 1s XPS spectra of CoP/g-C_3_N_4_ photocatalysts.

The chemical states of different elements in the prepared CoP/g-C_3_N_4_ samples were investigated according to the XPS spectra. As shown in Figure 3a, the peaks located at 799 eV and 797.4/781.8 eV were assigned to Co 2p_1/2_ and Co 2p_2/3_ in the CoP, which are closely related to Co^2+^ and Co^3+^ [26]. As shown in Figure 3b, the P 2p XPS spectrum for CoP was fitted into three peaks at 129.2 eV, 129.75 eV, and 133.45eV, attributed to the P 2p_2/3_, P 2p_1/2_, and P-O bond, respectively [27]. For the C 1s spectrum (Figure 3c), two characteristic peaks located at 284.8 eV and 287.98 eV corresponded to graphitic and N-C=N, respectively [15]. In the N1s spectrum (Figure 3d), four characteristic peaks located at 398.5 eV, 399.4 eV, 400.7 eV, and 404 eV could be attributed to C-N=C, N-(C)_3_, C-NH, and π excitation [28]. The result further confirmed the successful synthesis of the CoP/g-C_3_N_4_ photocatalysts.

**Figure 4:**
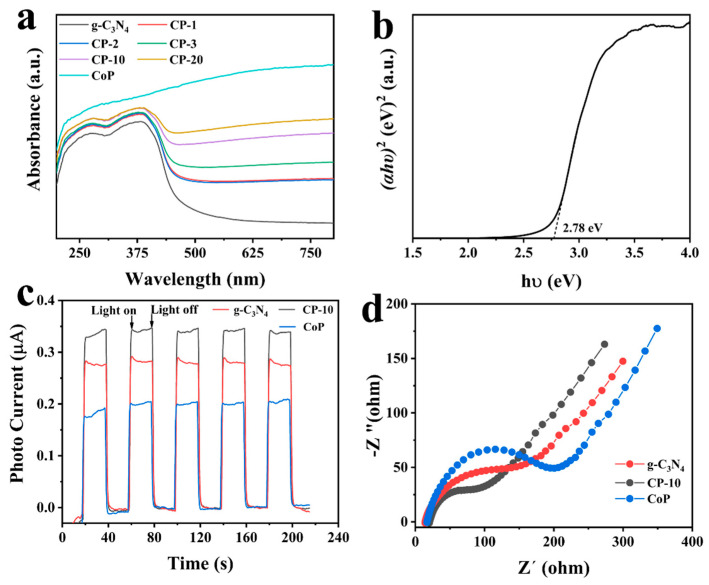
(**a**) UV-vis DRS, (**b**) band gap energy, (**c**) photocurrent transient responses, and (**d**) EIS Nyquist plots.

UV-vis DRS, transient photocurrent response curves, and EIS Nyquist plots were employed to examine the optical and photoelectrochemical properties. As displayed in Figure 4a, all prepared photocatalysts showed obviously visible light absorption. For pure g-C_3_N_4_, the absorption edge could be observed at about 460 nm, while CoP showed strong light adsorption in visible regions. Compared with pure g-C_3_N_4_, the absorption edges of composite photocatalysts showed a slightly red shift, which demonstrated that the presence of CoP led to an increase in the light harvesting ability. According to the Tauc plot in Figure 4b, the band gap energy of g-C_3_N_4_ was 2.78 eV, similar to that of other research [29]. Figure 4c shows the transient photocurrent response curves of g-C_3_N_4_, CoP, and CP-10. Among them, CP-10 had highest photocurrent density, suggesting high separation and transfer efficiency of photoinduced electron–hole pairs. As illustrated in Figure 4d, CP-10 showed the smallest semicircle of the arc radius in all EIS Nyquist plots, which was in accordance with the results of photocurrent response curves and further demonstrated the low resistance of charge transport.

### 3.2. Photocatalytic Performance

**Figure 5:**
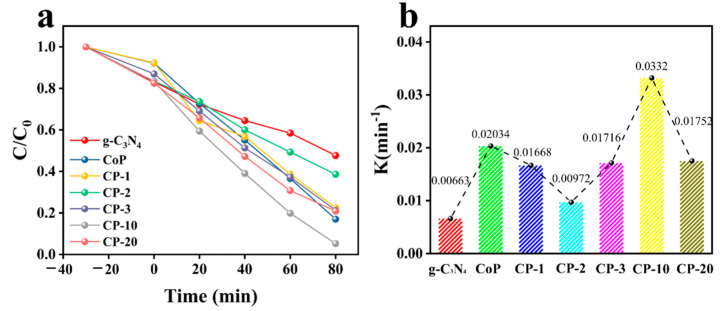
(**a**) Ammonia nitrogen removal rate of prepared photocatalysts and (**b**) apparent rate constants (k) for ammonia nitrogen removal rate with the prepared photocatalysts.

The catalytic activities of the prepared photocatalysts were evaluated according to the degradation of ammonia nitrogen. As shown in Figure 5a, under dark adsorption conditions for 30 min, the ammonia nitrogen removal rate presented an obvious improvement with the addition of composite photocatalysts compared with pure g-C_3_N_4_, demonstrating the outstanding adsorption capability of the prepared photocatalysts. Under light irradiation, a removal rate of about 52.3% for ammonia nitrogen in 80 min was observed with pure g-C_3_N_4_, while pristine CoP exhibited about 83% removal of ammonia nitrogen. The photocatalytic activities of all composite photocatalysts (CP-1, CP-2, CP-3, CP-10, CP-20) were higher than that of pure g-C_3_N_4_. CP-10 clearly exhibited the optimal removal rate (94.8%) for ammonia nitrogen of the prepared composite photocatalysts. This phenomenon indicated that an appropriate amount of CoP loaded onto the surface of g-C_3_N_4_ could contribute to improving the photocatalytic performance of the resulting photocatalyst. However, CP-20 obtained a 79.1% removal rate of ammonia nitrogen, which is lower than that of CP-10. The result demonstrated that an excessive amount of CoP reduces the photocatalytic performance of the resulting photocatalyst, which may be attributed to a decrease in the number of active sites due to the aggregation of CoP nanoparticles [30]. According to Figure 5b, the apparent rate constant (k) of CP-10 was 5 times higher than that of pure g-C_3_N_4_ and 1.6 times higher than that of pure CoP nanoparticles. The result indicated the excellent photocatalytic activity of CP-10 composite photocatalysts.

### 3.3. Effect of Initial Solution pH and Ionic Strength on the Removal of Ammonia Nitrogen

**Figure 6:**
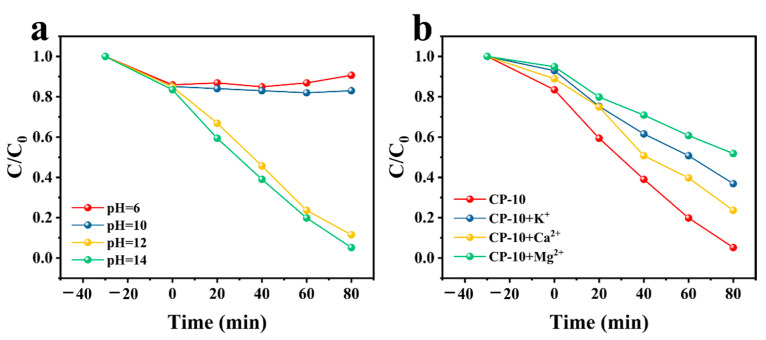
The influences of (**a**) pH value and (**b**) ionic strength on the removal of ammonia nitrogen.

As the initial solution pH and ionic strength might affect the removal of ammonia nitrogen in the photocatalytic process, they were also investigated in this research. As shown in Figure 6a, different removal rates of ammonia nitrogen were observed under different pH values (6, 10, 12, 14), where the removal rate of ammonia nitrogen increased with the pH within 80 min of the photocatalytic reaction. This phenomenon could be due to the change from NH_4_^+^ to NH_3_ under alkaline conditions, as the removal of NH_3_ is easier than NH_4_^+^ [5]. Nevertheless, the removal rate of ammonia nitrogen was approximately the same at pH 12 and 14, suggesting that higher pH values have an insignificant impact on ammonia nitrogen removal. The result is consistent with a previous report and might be caused by a decrease in the number of active sites on the photocatalysts [23]. Therefore, the initial solution pH was adjusted to 12 in this research.

As inorganic cations are ubiquitous in aquatic environments, their effects on the removal of ammonia nitrogen were also investigated in this research. As shown in Figure 6b, the existence of all tested cations (Na^+^, Ca^2+^, Mg^2+^) significantly prevented the removal of ammonia nitrogen, with the order of inhibition effects being Mg^2+^ > Ca^2+^ > Na^+^. The results might be attributed to two main reasons. On one hand, these cations could adsorb on the surface of synthesized photocatalysts, which might lead to a decrease in the number of catalytic active sites [31]. On the other hand, Mg^2+^ and Ca^2+^ could be transformed into Mg(OH)_2_ and Ca(OH)_2_ in alkaline solution, which would not only decrease the production of hydroxyl radicals (·OH) but also the solution turbidity, which creates unfavorable conditions for the photocatalytic reaction [5].

### 3.4. Mechanism of Ammonia Nitrogen Removal

**Figure 7:**
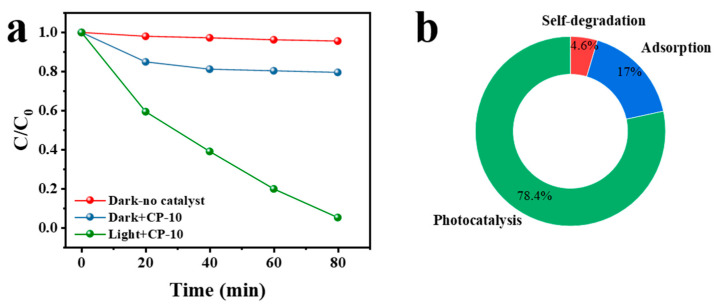
(**a**) Ammonia nitrogen removal under different experimental conditions and (**b**) percentage of influence of different experimental conditions for ammonia nitrogen removal.

Recent research has found that stripping, illumination, stripping, and photocatalysis could effectively enhance ammonia nitrogen removal [5]. In this research, these main influencing factors were classified as self-degradation, adsorption, and photocatalysis, which were tested through consideration of the following experimental conditions: dark with no catalyst, dark with CP-10 photocatalysts, and light with CP-10 photocatalysts. As shown in Figure 7a, the three experimental conditions showed different degrees of influence. According to the results, the percentages associated with the different experimental conditions for ammonia nitrogen removal were obtained through mutual subtractions. As shown in Figure 7b, self-degradation, adsorption, and photocatalysis had contributions of 4.6%, 17%, and 78.4% to ammonia nitrogen removal, respectively. Therefore, photocatalysis was clearly the most important controlling factor for ammonia nitrogen removal in this research.

### 3.5. The Mechanism of Ammonia Nitrogen Removal over CP-10 Photocatalysts

**Figure 8:**
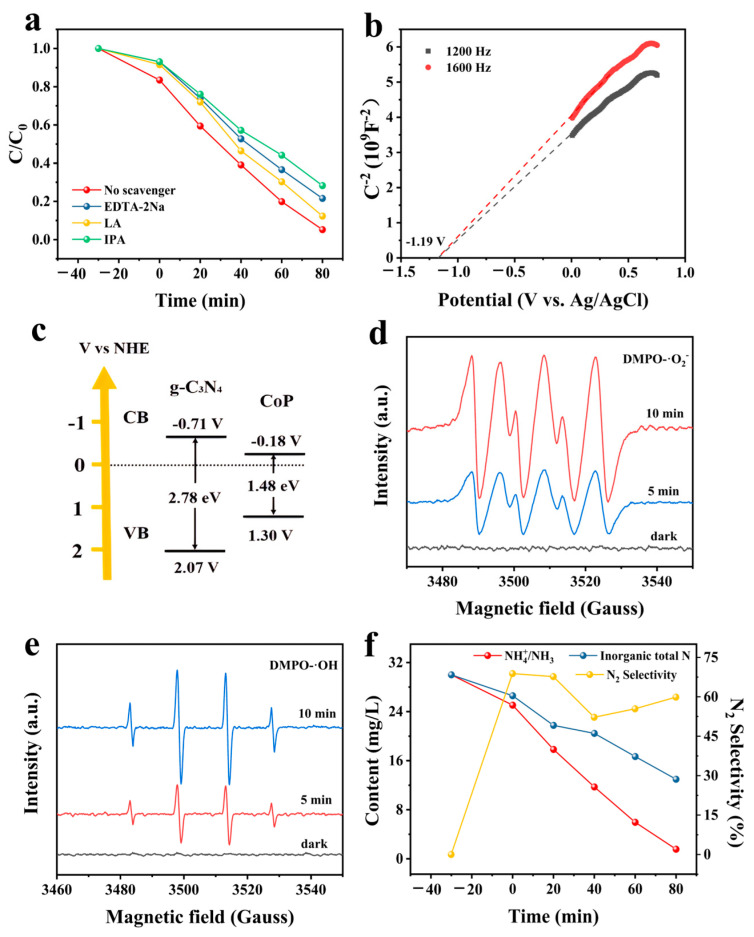
(**a**) The removal of ammonia nitrogen over CP-10 photocatalysts in the presence of various scavengers, (**b**) Mott–Schottky plot of CP-10, (**c**) schematic band gap structures of prepared photocatalysts, (**d**) TEMPO-h^+^ and (**e**) DMPO-·OH for S-CN samples, and (**f**) conversion of inorganic nitrogen in the process of ammonia nitrogen removal.

The mechanism of photocatalytic ammonia nitrogen removal is associated with reactive oxygen species (ROS), such as h^+^, ·O^2−^, and ·OH, as has been reported in previous research [32]. As shown in Figure 8a, ammonia nitrogen removal was obstructed with the addition of scavengers, indicating that h^+^, ·O^2−^, and ·OH had positive effects on the photocatalytic process. In particular, compared to h^+^ and ·O^2−^, ·OH radicals were found to play a main role in the algal.

Furthermore, the band gap structures of the prepared photocatalysts were analyzed in order to comprehend the production and transfer of ROS in the photocatalytic process. First, the Mott–Schottky plot methods were used to investigate the flat band of pure g-C_3_N_4_. As shown in Figure 8b, the flat band potential (E_fb_) was −1.19 V vs. the Ag/AgCl electrode, which could be converted to the conduction band (E*_CB_*) of −0.71 V (normal hydrogen electrode, NHE) using the following Equation [33].

***E_CB_*_(NHE)_ = *E*_fb(Ag/AgCl)_ + 0.059 × pH + *E*_(AgCl)_ − 0.1** (3)

where E(AgCl) = 0.197 V and the pH value was around 6.5. In view of the band gap energy and the CB position of g-C_3_N_4_, the valence band (VB) of g-C_3_N_4_ was 2.07 V. Furthermore, CB and VB values of CoP could be obtained in our previous report, which were −0.18 V and 1.3 V, respectively [22]. According to the above results, the band gap structures of g-C_3_N_4_ and CoP are shown in Figure 8c. Thus, ·O^2−^ could be generated in the solution due to the higher CB potential of g-C_3_N_4_ compared to O_2_/·O^2−^ (−0.33 V). Similarly, ·OH could also be generated in the solution because of the lower VB potential of g-C_3_N_4_ than that of OH^−^/·OH (1.99 V). The ESR experiments were further used to detect the generation of reactive species in the photocatalytic process. As shown in Figure 8d,e, the characteristic peak signal of DMPO-·O^2−^ adduct and DMPO-·OH adduct could be observed under light illumination, which demonstrated the formation of ·O^2−^ and ·OH through prepared CP-10 photocatalysts. The result was in accordance with the analysis of the band gap structures.

Generally, inorganic total nitrogen includes ammonia, nitrate, and nitrite [34]. In this research, the concentration changes of inorganic total nitrogen and ammonia nitrogen were measured in order to understand the nitrogen selectivity $S\left(N_{2}\right)$. As shown in Figure 8f, the concentration of ammonia nitrogen and inorganic total nitrogen significantly decreased with increasing photocatalytic time. According to the calculation, the $S\left(N_{2}\right)$ was about 60%, which might be enhanced continuously with increasing reaction time. The result indicated that ammonia nitrogen could be degraded through the photocatalytic reaction in the presence of CP-10 photocatalysts.

**Figure 9:**
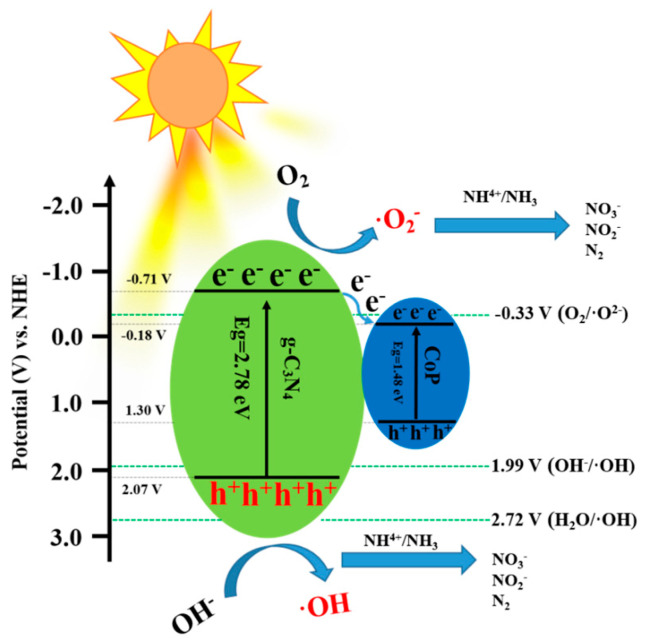
Schematic diagram of the possible photocatalytic mechanism of ammonia nitrogen removal.

According to the above analysis, a possible photocatalytic mechanism for ammonia nitrogen removal using the prepared composites was determined, as shown in Figure 9. During light exposure, the generation of photo-induced electron–hole pairs in both g-C_3_N_4_ and CoP takes place on the surface of photocatalysts. Moreover, the CB electrons of g-C_3_N_4_ can be transferred to the CB position due to the attraction of CoP semiconductors, and similar situations have been reported in our other study [22]. The separation efficiency of electron–hole pairs could be obviously enhanced in view of this phenomenon. In this way, more ·O^2−^ and ·OH could be generated through the redox reaction of the electron and the hole, which caused the efficient removal of ammonia nitrogen. It had been reported that the removal of ammonia nitrogen was influenced by several steps in the presence of active species, which were listed as follows [5,9]:

***g-C_3_N_4_/CoP + hv → e^−^ + h^+^,*** (4)

***O^2−^ + e^−^ → ·O^2−^,*** (5)

***h^+^ + H_2_O → ·OH,*** (6)

***NH_4_^+^/NH_3_ + O^2−^/·OH/h^+^ → NO_3_^−^/NO_2_^−^/N_2_,*** (7)

In the photocatalytic reaction, the active species (·O^2−^, ·OH and h^+^) excited by the as-prepared photocatalysts as the source of photocatalytic oxidation could transform ammonia nitrogen into NO_3_^−^, NO_2_^−^, and N_2_.

## 4. Conclusions

In this work, CP (g-C_3_N_4_/CoP) composite materials are synthesized through a simple self-assembly method and used in ammonia nitrogen pollution. Among them, CP-10 composites exhibited an outstanding removal rate (94.8%) for ammonia nitrogen within 80 min because of the heterogeneous structure caused the high separation and transmission of electron–hole pairs. Moreover, the oxidative products of ammonia nitrogen were mainly N_2_ with a small amount NO_3_^−^ and NO_2_^−^, which indicated that photocatalytic ammonia nitrogen removal was successful. This research has great significance for the application of photocatalytic technology for ammonia nitrogen removal.

## Data Availability

Data are contained within the article. The original contributions presented in this study are included in the article. Further inquiries can be directed to the corresponding authors.
